# Microplastic and Nanoplastic in Crops: Possible Adverse Effects to Crop Production and Contaminant Transfer in the Food Chain

**DOI:** 10.3390/plants13172526

**Published:** 2024-09-08

**Authors:** Bhakti Jadhav, Agnieszka Medyńska-Juraszek

**Affiliations:** Institute of Soil Science, Plant Nutrition and Environmental Protection, Wroclaw University of Environmental and Life Sciences, 53 Grunwaldzka Str., 50-357 Wrocław, Poland; bhakti.jadhav@upwr.edu.pl

**Keywords:** microplastic, plants, toxic compounds, food chain, crop production, plant pathogens

## Abstract

With the increasing amounts of microplastic (MP) deposited in soil from various agricultural activities, crop plants can become an important source of MP in food products. The last three years of studies gave enough evidence showing that plastic in the form of nanoparticles (<100 nm) can be taken up by the root system and transferred to aboveground plant parts. Furthermore, the presence of microplastic in soil affects plant growth disturbing metabolic processes in plants, thus reducing yields and crop quality. Some of the adverse effects of microplastic on plants have been already described in the meta-analysis; however, this review provides a comprehensive overview of the latest findings about possible adverse effects and risks related to wide microplastic occurrence in soil on crop production safety, including topics related to changes of pesticides behavior and plant pathogen spreading under the presence MP and possibly threaten to human health.

## 1. Introduction

Microplastics can enter soil through three main routes: air, water, or indented plastic deposition by humans. Agriculture activities strongly depend upon plastic. From farming tools, fertilizers, and mulches to storage systems and equipment used in agricultural practices plastic is present in whole crop production systems. In addition, agricultural soils receive a large number of microplastics (MP) from water irrigating systems, airborne pollution, and sewage sludge application [1]. Although soils are considered one of the most important sources of MPs in the food chain [2], terrestrial ecosystems are receiving much less attention from scientists, and the risk is related to the presence of micro- and nanoplastics (MPs/NPs) in agricultural soils. The possible impacts of MPs/NPs on soil productivity and crop production are not well defined and the number of studies in this field is still insufficient. However, there is a lot of contradictory information indicating the significant impact of microplastic particles on plants and crop production. Poor mobility and relatively large size of particles minimize the risk of microplastic contaminants uptake by plants. However, nanoplastics may be a problem due to more and more evidence that NPs can be absorbed by plants through various mechanisms [3,4,5]. MPs can be adsorbed onto roots affecting plant growth [6,7] and inducing a widespread toxic effect on many physiological and biochemical processes in plants, such as delaying or reducing seed germination, inhibiting plant growth, changing root traits, reducing biomass, delaying and reducing fruit yield, interfering with photosynthesis, causing oxidative damage, and producing genotoxicity [3,8,9,10]. MPs enter soil having different origins, chemical composition, shapes, sizes, or surface properties and are subsequently varied in their physical and chemical properties leading to unpredictable effects on crops and soil ecosystems [9]. There is still limited evidence showing how MPs could affect the physical, chemical, and biological properties of soil, as our knowledge is mainly based on experiments run under controlled conditions e.g., pot or incubation data. There is a lack of observations in the field as microplastic identification in soils is still challenging. MP particles affect soil porosity, water holding capacity, size and formation of aggregates, pH, carbon pools, and nutrient availability [7,9,11,12]. Additionally, MPs can sorb toxic compounds acting as vectors of organic and inorganic contaminants becoming easily transferable in the food chain. One of the biggest uncertainties is how much MPs/NPs are accumulated in the soil, as consequently to other well-known contaminants in soil, the dose-response in plants can be different. Therefore, predicting the influences of MPs on soil properties, as well as recognizing the long-term effects on plant physiology and crop production should be a research priority. Microplastics absorbed by plants can enter higher food chain trophic levels, which may ultimately pose risks to human health. Whether MPs/NPs can be taken up by plants and what are the mechanisms behind this process is still a controversial topic. However recent studies from the last two years showed evidence that plastics can be taken up by plant roots under certain conditions including properties of soil, physiological characteristics of plants e.g., transpiration rate, or even plastic type and properties e.g., particle size and surface charge [10]. Most of the published research focused only on one type of polymer—polystyrene and there is a lack of knowledge on other important polymers most commonly occurring in soil e.g., polyesters or polyethylene.

This review aims to summarize the latest findings about the impacts of microplastic/nanoplastic on plant physiology, crop production safety, and the transfer of potentially toxic compounds and pathogens from soil to plants and the food chain.

## 2. Materials and Methods

In the current review paper, all materials were obtained from scientific databases available online. To obtain more comprehensive data, multiple databases such as Web of Science, Science Direct, Nature, Springer, Google Scholar, and PubMed were used for sourcing information. The topics highlighted in this review paper were searched based on the keywords “microplastic”, “nanoplastic”, “plants”, and “food chain”. Not all relevant literature could be found by these keywords and phrases were used for more advanced search e.g., “microplastic uptake by plants”, “impact of micro/nanoplastic on plants”, “microplastic as a vector of contaminants”, “plant tolerance”, “microplastic and antibiotics”, and “microplastic and pathogens”. For the review preparation, only the latest findings on the topic and research papers from 2019 to 2024 were considered. Approximately 130 recent articles were downloaded and based on the abstract grouped into five sets of literature data: (1) Micro- and nanoplastic uptake by plants, (2) microplastic effects on plant growth and plant stress, (3) direct and indirect effects of microplastic on nutrient availability through nutrient cycling changes, (4) an effect of microplastic on other soil contaminants bioavailability to plants, and (5) microplastic, antibiotics resistance, and plant pathogens spread.

## 3. Microplastic Uptake by Plants

The barriers to entry of MPs into plants include chemical and physiological barriers. It has been assumed that MPs are too large to pass through the physical barriers of intact plant tissue and hence cannot be internalized directly into plant tissue [4,13,14]. The plant epidermis and cell wall make it difficult for larger microplastics to penetrate the tissues of vascular plants, although NPs can penetrate plant cells directly [13]. The thickness and architecture of these barriers vary with species, growth stages, and environmental conditions. Mostly reported in the literature limit for the plant to take up a molecule is 20 nm. Large nanoparticles (>100 nm) are generally taken up by plants despite smaller molecules (<20 nm) [3,15]. The uptake of MPs can be tracked with many different advanced tools, including laser confocal scanning microscopy, optical microscopy, spectroscopic techniques, or Py-GC/MS to quantify the amounts of plastic accumulated in plant tissues [16,17]. Recently coherent anti-Stokes Raman scattering (CARS) and stimulated Raman scattering (SRS) imaging, were promoted as new methods of tracking MPs/NPs uptake by plant cells and it was indicated that polystyrene particles with a diameter of less than 2 μm in savoy cabbage (*Brassica oleracea* var. *sabauda* L.) were taken up by seedlings, affecting shoot and root biomass of the plant [18]. These tools improved our stage of knowledge about microplastic presence in plants and showed that MPs, or more likely NPs, can accumulate in different plant tissues that are connected by the vascular system. Different transport mechanisms from roots to shoots and leaves have been indicated; however, endocytosis, apoplastic transport, entry through cracks, and entry through leaves stomata are the most probable pathways of MPs to plant tissues [13,19]. Researchers hypothesized that the nanoparticles in root tip mucilage enhance recognition of the cell membrane, which is highly unstable due to ongoing cell division. The epidermal layers of complete apical root sections are the only sites where NPs can penetrate the apical meristem, as Kasparian is still developing [14]. The surface charge is an important factor because nanoplastics with different surface charges cause different phenomena in the absorption process [20]. Sun et al. [21] suggested that nanoplastics with negatively charged surfaces might be absorbed by the root hairs in the maturation region and internalized into the stele via the apoplastic pathway. Apoplast transport is also considered to be a way for roots to absorb NPs. NPs can be internalized from the root epidermis to the cortex through the apoplast pathway, and even reach the xylem vessels [16]. Most of the micro/nanoplastics are adsorbed to the root surface due to the strong adsorption of root mucus and the hydrophobic interaction of plastic particles with the cell wall. Jiang et al. [3] observed the presence of MPs in roots suggesting that particles can be trapped in cell wall pores and transported through the vascular system, xylem, and phloem with water and nutrient flow. Due to limited diffusion through the cell wall pores, microplastic with a size of 1 mm cannot penetrate the cell wall [19]. Cell wall pores are not the only path for nanoplastics to penetrate plant tissues. Evidence shows that cracks in the lateral roots can also act as a pathway [20,22]. This way of plastic transfer was indicated for wheat (*Triticum aestivum* L.) and lettuce (*Latuca sativa* L.). Li et al. [23] described the crack-entry pathway providing evidence in support of submicrometre- and micrometre-sized polystyrene and polymethylmethacrylate particles penetrating the stele of both species using the crack-entry mode at sites of lateral root emergence. The plastic particles were subsequently transported from the roots to the shoots. Although the root pathway is the main route for plastics to enter the plant, the leaf surface should not be omitted in this process. Airborne micro/nanoplastics can accumulate on the leaf surface through atmospheric deposition and they can become physically trapped in leaf structures such as trichomes, stomata, and cuticles [16,24]. Sun et al. [25] showed that polystyrene particles can effectively accumulate on the maize leaves, accompanied by observable particle aggregation, and enter mainly through the stomatal opening and move down to the roots through the vascular bundle. Moreover, in the same study, it was proven that plastic particles can be transferred down to roots; however, this transfer depends on particle size as plastic particles need to pass a lot of physiological barriers moving downward. There is some evidence that some species have some mechanisms preventing the transfer of MPs/NPs particles to above-ground parts. For example, in lettuce (*Latuca sativa* L.), barley (*Hordeum vulgare* L.), and carrots (*Daucus carota* subsp. *Sativus*) microplastics accumulated mainly in the vascular systems of roots and stems, while in maize (*Zea mays* L.), wheat (*Triticum aestivum* L.), or thale cress (*Arabidopsis thaliana* L.) plastic particles are transferred from roots to shoots via vascular system [12,20,21,26]. In cucumber (*Cucumis sativus* L.) plants, polystyrene MPs of 100 to 70 nm size were absorbed by the roots and transported to the above-ground parts of the plant including leaves, flowers, and fruits [17,27]. Another study showed that the intensity of MPs/NPs uptake depends on the growth stage of the plant. Studies on rice (*Oryza sativa* L.) indicated that more plastic particles are taken up in the early stage of plant growth, until grain-setting than in the mature phase when rice grains our in the milky phase [28].

## 4. Microplastics Affect Plant Growth by Inhibiting Plant Metabolism

MP stress reduces plant growth and development by altering several key physiological processes including ionic homeostasis, redox regulation, and photosynthesis [29]. Information on the effects of MP on plant growth and physiology is still quite limited. MPs adsorbed on the surface of plants may inhibit photosynthesis, possibly reducing crop growth [30]. The morphological characteristic of microplastic particles e.g., size, shape, polymer chemical composition is an important factor [31]. Most of the published research describes one type of polymer—polystyrene (PS); however, some researchers claim that polyvinyl chloride (PVC) is much more toxic to plants [32]. From all the characteristics the particle size seems to be the most important as only MPs with a size < 100 nm are available for plants. For instance, MPs with a particle size of 100 nm reduced wheat (*Triticum aestivum* L.) seed germination by a relatively higher percentage when compared with MPs with a particle size of 5 µm [33]. Also, the microplastic dose is important showing linear dependency and more adverse effects with increasing doses of MP in soil. A dose-dependent phenomenon was observed in a study on lettuce (*Lactuca sativa* L.), wherein the weight, height, root length, and leaf number decreased with increasing microplastic concentration in soil [34]. MP presence can affect seed germination and seedling growth; however, this phenomenon is not always indicated in these studies. Giorgetti et al. [35] observed no statistically significant differences in germination percentage of onion (*Allium cepa* L.) between the treatments with increasing doses of polystyrene. While root-length reduction and cytological analysis of the root meristems indicated cytotoxicity (reduction in mitotic index) and genotoxicity (induction of cytogenetic anomalies and micronuclei) starting from the lowest dose. Similar findings were observed on faba bean (*Vicia faba* L.). Exposure to 5 µm PS-MPs caused a significant decrease (*p* < 0.05) in root length and relative root elongation only at higher concentrations of 50 and 100 mg L^−1^, although bigger MP particles (100 nm) had no negative effect on root elongation [3]. An opposite effect was observed on three ornamental plants: white clover (*Trifolium repens* L.), Chinese violet cress (*Orychophragmus violaceus* L.), and garden balsam (*Impatiens balsamina* L.), where the seed germination rates and germination potential were significantly reduced in the presence of polystyrene MPs with size 100 nm [36]. In another study, after 30 days of exposure to both conventional microplastics (like polypropylene and polyethylene) and biodegradable microplastics (such as polyhydroxybutyrate and polylactic acid), perennial ryegrass (*Lolium perenne* L.) showed a 35–71% reduction in root and shoot biomass, as well as chlorophyll content [37]. Similarly, the black gram seeds (*Vigna mungo* L.) germination rate reduced from 83.33% to 66.67% after 24 h of exposure to 1% polyethylene MPs of 60-micron size [38]. However, the same study reported minimal effect of MP exposure on tomato (*Solanum lycopersicum* L.) seed germination. The slight decline in the rate of increase in root length in the first 24 h was reversed after 48 to 72 h of exposure and the root growth rate was the same as the control. The study concluded that the effect of MPs on black gram (*Vigna mungo* L.) and tomato (*Solanum lycopersicum* L.) seed germination and initial growth rate declined over time. Such that the longer the seeds were exposed to the MPs the more tolerant they became, as far as germination is concerned. A summary of the adverse effects of MP/NP on different crop germination and early stages of development has been shown in Table 1. One of the probable mechanisms behind the inhibition of seed germination in the presence of MP particles is competition for water as MPs can accumulate on the surface of germinating seeds and obstruct water uptake [22]. There is also some evidence showing that aboveground parts of plants can be impacted by the presence of microplastics in soil. Nanoplastics can affect the sugar and protein metabolism of plants. Li et al. [39] described that the biomass of cucumber (*Cucumis sativus* L.) plants significantly decreased after exposure to 300 nm polystyrene NPs. Similarly, the chlorophyll a, chlorophyll b, soluble sugar, carotenoid, and proline content, as well as the fluorescence of cucumber (*Cucumis sativus* L.) leaves were significantly reduced by 100 nm NPs. Qi et al. [40] reported that 10 g kg^−1^ MPs had adverse effects on both root and shoot systems of wheat (*Triticum aestivum* L.), causing negative effects on both vegetative and reproductive growth.

## 5. Microplastic Causes Plant Oxidative Stress Reducing the Nutrition Value of Crops

Antioxidant enzyme activity catalase (CAT), superoxide dismutase (SOD) and peroxidase (POD), and oxidative root tip cells are the most commonly used plant stress indicators and antioxidant defenses induced by the presence of toxic or disturbing compounds [35]. It is common to indicate increased activity of oxidative stress markers giving evidence of stress induction in plants when microplastic is present in the growing medium [22]. A plant’s oxidative status is dependent on the production of reactive oxygen species (ROS). The major species of reactive oxygen include superoxide anion (O_2_·), hydrogen peroxide (H_2_O_2_), and hydroxyl radical (·OH) [11,31]. A low concentration of microplastics enhances the expression of ROS-stimulating enzyme-coding genes, thereby increasing the activity of various antioxidant enzymes [20], while high concentrations of MPs may mobilize antioxidant defense system and make plants more resistant to plastic stressors [1,2,15,33,36,41,42]. POD gene in cucumber (*Cucumis sativus* L.) leaves significantly increased after cucumber (*Cucumis sativus* L.) plants were exposed to 100, 500, and 700 nm NPs. The increase in the POD gene can be correlated with the size of NPs particles and in 700 nm NPs treatment was almost 50 times higher than in control and small particle size treatments [39]. Similarly, findings were observed for ROS and the enzymes in the carbohydrate metabolism of barley; however, in the study, it was also indicated that roots and leaves can be impacted differently [43]. Studies on faba beans (*Vicia faba* L.) showed increased antioxidant enzyme activity and oxidative damage of root tip cells in the presence of polystyrene molecules, even at the lowest concentration (50 mg L^−1^). Pignatelli et al. [32] showed that garden cress (*Lepidium sativum* L.) is exposed to chronic stress when MPs are present in soil and this can be observed in chlorophylls, carotenoids, aminolaevulinic acid, and proline production. Giorgetti et al. [35] observed an oxidative response in increased H_2_O_2_ levels in onions, suggesting that the effect of MPs/NPs depends on the dose (only in treatments with the highest dose oxidative stress was observed) and surface charge. In *Arabidopsis*, an increase in H_2_O_2_ and O_2_· levels in response to polystyrene NPs was noted, where the positively charged NP (PS-NH_2_) induced stronger H_2_O_2_ accumulation than the negatively charged ones (PS-SO_3_H) [21]. Arikan et al. [44] proved that polystyrene nanoplastic contamination increases ROS accumulation in wheat (*Triticum aestivum* L.), decreasing photosynthesis, and reducing growth and water content in wheat (*Triticum aestivum* L.) leaves. Wu et al. [45] indicated multi-pathway negative effects on rice (*Oryza sativa* L.) exposed to polystyrene MPs. Over 70% of the metabolites, including 12 amino acids, 16 saccharides, 26 organic acids, and 17 others (lipids and polyols) significantly decreased with the increase in PS-MP doses. It was also observed that the rice (*Oryza sativa* L.) leaves exposed to 500 mg L^−1^ of PS-MPs showed significantly lowered biomass and poorer growth conditions than those that were exposed to 50 mg L^−1^ and 250 mg L^−1^ of PS-MPs. In maize (*Zea mays* L.) exposed to polystyrene nanoparticles, up to a 4.5-fold increase in malondialdehyde (MDA) and up to 1.53-fold increase in superoxide dismutase and glutathione-peroxidase activities in the roots were observed. The level of ROS activity is correlated with the size of the NPs [46]. The study showed that the presence of polystyrene NPs in the soil resulted in oxidative stress to the plant as a response to the presence of toxins significantly interfering with the metabolism of maize (*Zea mays* L.) plants. Similar findings were observed on other crops, suggesting the photosynthesis function of plants can be affected by MPs. Rice (*Oryza sativa* L.) plants grown in soil contaminated with MPs had 35.45% empty shells and the peanut seeds weighed 3.45% less when grown in MPs-containing soil [28]. Evidence has also been provided to show that the quality of the crops is also reduced by the presence of MPs in the soil. Nutrients such as amino acids, unsaturated fatty acids, and minerals can be significantly reduced when the plants are exposed to MP [28]. Moreover, MPs can accumulate in grains, disturbing mineral elements homeostasis, and in the presence of 250 mg kg^−1^ polystyrene (80 nm), the contents of Ca, Mn, and Zn in rice (*Oryza sativa* L.) grains decreased by 16.33%, 16.86%, and 9.94%, respectively, compared with those of the control treatments, with no significant effect on yields [26], but relevant changes of grain quality and nutrition facts. Microplastic can affect nutrient uptake by plants; however, the effect can be different depending on the element, plant species, and plant phenotype. Studies on tomatoes (*Solanum lycopersicum* L.) showed that Cu concentration in the presence of polypropylene particles increased in shoots, while nitrogen and phosphorus uptake significantly decreased [47]. Several studies have reported that MP addition into the soil could have an impact on the N cycle at different levels; altering the microbiota and the abundance of genes, and therefore, the enzymes that catalyze the different stages of the N cycle [48,49]. Fei et al. [50] indicated that some groups of bacteria e.g., *Acidobacteria, Bacteroidetes, Gemmatimonadetes*, and *Proteobacteria* responsible for N processing have significantly more abounded in soils with microplastics. Rong et al. [48] indicated that some bacterial communities of denitrifies like *Pseudomonas, Streptomyces,* and *Methylobacterium* can be enriched in soils with LDPE amendment, thus affecting nitrogen cycling processes. Microplastics alter soil N cycling, however, impact nitrogen metabolism and protein production by plants which are commonly indicated. High concentrations of polypropylene (1% *w*/*w*) inhibited N uptake in peanut (*Arachis hypogaea* L.) plants by damaging root cells and disturbing soil N cycling [51]. The results of Ingraffia et al. [52] showed that the presence of microplastic fibers in soil limited maize (*Zea mays* L.) growth and N uptake by 30%, increasing nitrogen loss via leaching. Zhang et al. [53] stated that PVC microparticles can affect soil enzyme activity, microbial biomass, and nitrogen metabolism in wheat (*Triticum aestivum* L.). Different type of plastics affects C and N cycling differently and potentially biodegradable plastics can promote protein synthesis in plants, likely due to C release during decomposition, while non-biodegradable plastic fractions suppress the N transformation in plant and soil [54]. Shi et al. [55] showed that MP presence facilitated the activity of urease and nitrate reductase in soil. Therefore, the introduction of MPs in soil could result in an elevation of NH^4+^ levels [56].

## 6. Microplastic Aging in Soil Enhances Contaminants and Pathogens Adsorption

Microplastic in soil undergoes photodegradation, weathering, and oxidation processes in the soil leading to surface changes and behavior of material called “plastic aging”. Recent research shows that pristine and aged MPs may have distinct effects on plant and soil microbial activity [57,58,59,60]. Changes in surface properties are important in defining the tendency of MPs to form aggregates, as well as to transport various chemical species in the environment [57] or water absorption. The surface of aged alkyl polymers (i.e., PE and PP) is characterized by enrichment in oxygen-containing functional groups (especially carbonyl and hydroxyl groups), increased hydrophilicity, and a shift toward a more negative surface charge in comparison with pristine polymers [58]. This can, in turn, affect their potential toxicity. Exposure to 0.1% pristine PE was found to promote corn growth, while aged PE hindered its growth [59]. The mechanism behind this process can be observed in cellular response. Increased cytotoxicity of aged MPs and higher formation of ROS compared to pristine MPs was observed in cell cultures [61]. The aging process leads to a change in microplastic particle shape. Compared with pristine microplastic, aged materials have rough surfaces, and more cracks have some oxygen-containing functional groups that make them adsorb organic and inorganic pollutants [62] more efficiently. Depending on the shape of the particle, some particles can be easily attached to root hairs and transferred to plants [60]. Both conventional and biodegradable plastics often contain additives in the form of plasticizers, antioxidants, stabilizers, and pigments that are integrated into the polymeric matrix during the manufacturing process to improve their functionality [63]. Aging processes promote changes in plastic surface properties, leading to increased release of different organic compounds from plastic surfaces. Biodegradation products such as dodecanal are directly toxic to plants [40]. Bioplastics are widely regarded as more environmentally friendly options in various applications. This has for example led to some farmers choosing agricultural products such as mulch, seed coatings, or fertilizers made from biodegradable plastics such as polylactic acid (PLA) over those made from non-biodegradable plastics such as polyethylene (PE). However, recent studies show that MPs of biodegradable plastics can have a more detrimental effect on the chemical composition and microbial community in the soil than non-biodegradable plastics. PLA MPs biodegrade over a shorter period and cause an increase in the carbon composition of the soil. This reduces the microbial diversity in the soil resulting in more nitrogen being depleted from the soil. The altered soil composition favored the growth of certain species of microbes thus changing the microbial community and reducing biodiversity in the soil [54]. This ultimately resulted in poor growth of wheatgrass (*Triticum aestivum* L.) [63]. MPs have been shown to serve as surfaces for toxic elements such as polycyclic aromatic hydrocarbons, trace metals, herbicides, antibiotics, and polychlorinated biphenyls [64]. MPs in soil become a vector of potentially toxic compounds, which can be delivered to plants with plastic nanoparticles [9,44,64,65].

## 7. Microplastic in the Soil Increases the Bioavailability and Toxicity of Heavy Metals in Plants

Heavy metal response to microplastic addition to soil depends on both metal characteristics and plastic properties. Microplastic affects heavy metal speciation in the soil through different mechanisms: changing soil properties, direct adsorption on plastic surface, and transformation of HM from bioavailable to more stable organic-bound fractions [65]. Dissolved organic matter and pH are important factors affecting metal speciation and being affected by microplastic occurrence in soil [66]. High pH causes soil particles and MPs to have more charged sites on their surfaces, which effectively promotes the adsorption of HMs [66]. Different MPs have different potentials to modify HMs mobility in soil; however, currently, most of the published papers focused on relations between Cd and MPs, secondary Pb, and much less is known about As or Zn. This hypothesis might not apply to all metals and metalloids. A study by Zou et al. [67] showed that MPs interfered with the inhibitory effect of Cd on the taproot length and plant height of black nightshade (*Solanum nigrum* L.) LDPE-Cd^2+^ compound pollution inhibited the fresh weight of roots in the early growth stage of seedlings. Roy et al. [68] indicated that the presence of microplastics increased toxicity, decreased tolerance for stress, and enhanced uptake of Cd by red amaranth (*Amaranthus tricolor* L.). Meta-analysis performed by An et al. [69] showed that MPs enhanced the bioavailability of Fe, Mn, and As more significantly than Cu, Pb, Cd, and Zn. Microplastics have no discernible impact on Zn and As, but they can improve the bioavailability of Cu, Pb, Cd, Fe, and Mn, particularly Fe and Mn. In the same meta-analysis, it was stated that PE, PET, PP, PS, PU, PLA, and PES might improve the HM’s bioavailability, whereas PA, PBS, PHB, and PES had no impact. Microplastic can compete with soil constituents for sorption sites, thus HM speciation and sorption in the presence of MP particles in the soil might be different from unpolluted soils. One of the recognized mechanisms of HM adsorption on MPs is surface charge. Polar functional groups and the electrostatic interactions between plastic and metal cations may increase the bioavailability of potential plant compounds. This means that MPs/NP’s presence can alleviate the effect of heavy metal stress on plant root systems. Studies on maize (*Zea mays* L.) showed that polyethylene applied to soil alone has no significant phytotoxic effect on plant growth, however, co-presence of MPs and Cd amplified Cd phytotoxicity to maize (*Zea mays* L.) [70]. The coexistence of metals and microplastics in soils may pose risks to soil, plants, and even human health. With the increasing accumulation of MPs in soil, the risk of metal bioavailability enhancement also increases. Bethany and Golia [71] showed that with increasing doses of MPs in soil (2.5% and 5% *w*/*w*) the bioavailability and uptake of Zn and Cd by lettuce (*Latuca sativa* L.) also increased; however, Zn was more impacted and the content of this element in lettuce leaves increased by 21.5% after application of 5% *w*/*w* of PE-MPs. The results showed that MPs and Cd affected strawberry (*Fragaria x ananassa* L.) plant growth, plant biomass, the number of fruits, and root characteristics and increased the accumulation of Cd in the roots [72]. The promoting effect of MPs on HM accumulation is highly dependent on the type and particle size, potentially influencing plant adsorption. Among MP types, PE showed a higher promoting effect on Cd accumulation, with a mean increase of 29.4% [73]. However, some researchers claim that this chemisorption mechanism is beneficial for mitigating the toxic effect of copper and cadmium on plants by decreasing bioavailability and free fractions of these metals in soil solution. The accumulation of metals in wheat (*Triticum aestivum* L.) seedlings decreased by the presence of PS microplastic [74]. Polystyrene particles also mitigated Cd phytotoxicity and reduced uptake by Chinese cabbage (*Brassica chinses* L.) [75]. De Silva et al. [76] demonstrated that the presence of MPs reduced the toxic effect on lentils (*Lens culinaris* L.). There are more concerns about co-presence in soil of different groups of contaminants. Imran et al. [77] showed the adsorption of antibiotics and heavy metals on MP surfaces can expose bacterial pathogens to higher concentrations of these factors, leading to cross-resistance (resistance to antibiotics and metals) or co-resistance (resistance to multiple antibiotics) which should considered as an emerging health risk.

## 8. Microplastic Affects the Uptake of Organic Compounds by Plants Causing Higher Toxicity and Bioaccumulation in Crops

Microplastic particle adsorbs polycyclic aromatic hydrocarbons and participates in PAH transfer to plants. Abbasi et al. [78] described that all PAHs can be adsorbed on MPs with different strengths, showing the differences between different chemical characteristics of the components e.g., naphthalene is much easier adsorbed on PET-MP compared to phenanthrene. The results showed that MPs decreased the uptake of Phe in (*Glycine max*. L.) roots and leaves. MPs inhibited the accumulation of Phe in soybean (*Glycine max*. L.) roots and leaves. Micron-size MPs showed a higher inhibition of Phe uptake in roots than nano-size MPs. The combined toxicity of micron-size MPs and Phe to soybean (*Glycine max*. L.) plants was higher than that of nano-size MPs and Phe, and 100 μm MPs and Phe co-contaminant show the highest toxicity to soybean (*Glycine max*. L.). The activities of antioxidative enzymes and their gene expression showed that micron-size MPs induced higher genotoxic and oxidative damage to soybean (*Glycine max*. L.) roots than nano-size MPs, which decreased the activity of roots, thus leading to the lower uptake of Phe by soybean (*Glycine max*. L.) roots and leaves [79]. Liu et al. [80] described that the combination of PE-MP and phenanthrene causes oxidant stress, reduction in photosynthesis, and damage to wheat (*Triticum aestivum* L.) roots. The adsorption capacity of phenanthrene depends on the polymer type, the highest is for polystyrene, followed by polyethylene and polyvinyl chloride [81]. Microplastics also have adsorption for another group of toxic compounds commonly occurring in agriculture soils—pesticides. When MP undergo oxidation in soil, they become more efficient in pesticide binding, which means that chemical behavior and bioavailability of pesticides in the co-presence of MPs can be modified and unpredictable. Microplastic aging, the layered structure of the material, high porosity, and cracking form larger surface areas and more O-containing functional groups, such as carboxyl and hydroxyl groups. MPs with small sizes can absorb more pollutants than those with large sizes [82]. Behind the adsorption of pesticides different mechanisms compared to heavy metals can be indicated. Electrostatic forces, surface charge, or chemisorption play a secondary role compared to hydrophobic partitioning and formation of H-bondings between pesticide and O-containing functional groups on microplastic. MPs derived from agricultural PE films can absorb pesticides more easily. In general reduction in the sorption of pesticides on soil constituents in competition to sorption sites present at microplastic may increase the mobility and bioavailability of pesticides and other organic xenobiotics in soil [62,64]. However, the types of pesticides are so different, and their adsorption behaviors and mechanisms on microplastics are different [62]. Pesticide sorption depends on pH and as it was previously shown in our research [66], under the presence of microplastic soil pH may increase, thus sorption of pesticides should be decreasing. In batch experiments with pesticide sorption on different types of MPs, the effect was opposite to our hypothesis, showing that adsorption of five different pesticides Carbendazim (CAR), Dipterex (DIP), Dichlorovos (DIC), Diflubenzuron (DIF), 100 Malathion (MAL), Difenoconazole (DIFE) increased with increasing solution pH spiked with microplastics [83]. Comparing the sorption of pesticides in soil and microplastics, the influence of pH is less pronounced on sorption by MPs than on soil, this can be explained by the fact that the sorption of pesticides is driven by different mechanisms, mainly non-specific van-der Waals interactions [84]. The study of Sahai et al. [85] showed that sorption and vector effect on the transfer of pesticides and polycyclic aromatic hydrocarbons in soil polluted with plastic mulch films can be even 70% higher (depending on tested pesticide) compared to control fields without plastic mulch use. Higher pesticide adsorption is usually reported for smaller plastic particles, probably because of an increment of available adsorbent surface and effective sorption sites [86]. In soil dissolved organic matter (DOM) can compete for sorption sites with microplastics. Low DOM in the soil will promote pesticide sorption onto MPs. Due to the wide use of glyphosate, there is an urgent need to study the behavior and persistence of this pesticide in the presence of microplastics in soil. A study by Yang et al. [87] indicated that there is no significant impact of microplastic on glyphosate half-life. However, soil respiration rate and microbial activity were impacted by the co-presence of glyphosate and microplastic in the soil. In an eco-test, glyphosate and microplastic in a water solution increased the ed mortality of *Daphnia magna* by 23.3% after 7 days of exposure [88]. It was recognized that polystyrene microplastics and glyphosate had toxic effects on *Salvinia cucullata* L. causing physical damage and oxidative stress [89]. Studies on plant pesticide accumulation in the presence of microplastic are rare, however, there is an urgent gap in knowledge that should be filled in, as some researchers claim adverse effects of the co-presence of MP and pesticides in soil. Ju et al. [56] showed that bioaccumulation of pesticides (chlorpyrifos (CPF), difenoconazole (DIF) and their mixture) in radish (*Raphanus sativus* L.) exposed also to MP, increased and reduced the biomass of roots by 44%. Bioaccumulation of pesticides in edible crop parts is one the most alarming threats of microplastic occurrence in soil impacting human and animal health. There is also doubt if microplastic in soil and water should not be considered as a factor modifying the effect of pesticides on the target organism and its persistence in plants and soil.

## 9. Microplastics Can Cause Antibiotic Resistance and Act as Vectors of Plant and Foodborne Pathogens in the Food Chain

Antibiotics and microplastics are two relatively new groups of emerging pollutants, whose co-existence in the soil environment brings undefined risks to human and soil health. Interactions between microplastics and antibiotics are widely studied in aquatic systems and sewage sludge; however, due to difficulties in antibiotic identification in soil studies, the impact of microplastics on antibiotic resistance and accumulation in soils is limited. The probable mechanism of antibiotic adsorption on MPs is similar to other described in this review compounds—electrostatic attraction, chemisorption, and van der Walls forces are the processes involved in the sorption/desorption processes of antibiotic molecules on MPs in aquatic systems. However, the presence of organic matter and clay minerals, changes in pH, and bioaccumulation in soil microbes modify antibiotic behavior in soil. Recently it has been shown that MP presence in soil has a significant impact on the amount of antibiotics adsorbed on soil solids by providing additional binding sites or altering soil characteristics e.g., pH and dissolved organic carbon. The available antibiotics significantly decreased after the addition of three forms of plastic (PE, PVC, PP) to the soil; however, the decrease depended on soil characteristics [90]. Microplastic weathering and oxidation enhance the adsorption of antibiotics, increasing the electrostatic interactions and H-bonding [91], thus microplastic particles can act as vectors of antibiotics in soil. Scientists revealed that in an aquatic environment, microplastics can adsorb antibiotics (sulfadiazine, ciprofloxacin, amoxicillin, trimethoprim, and tetracycline) on their surfaces which results in their long-range dispersion and entry into the food chain [77]. Antibiotic resistance in bacteria, including foodborne pathogens, poses a major global health threat by rendering antibiotics ineffective for treating infections [92]. Agriculture further contributes to the problem by applying antimicrobial compounds in animal farming and application of organic amendments like manure or sewage sludge to soil. The wide occurrence of MPs/NPs and the potential acceleration of microbial interactions on plastic surfaces can implicate another problem—antimicrobial resistance (AMR). One of the biggest concerns is that microplastics in agricultural soils become hot spots for antibiotic resistance genes (ARGs). ARGs entering agricultural soil can pose a threat to human health in many ways, such as through direct contact or absorption by plants into the food chain. Therefore, agricultural soil is suspected to be an important source of antibiotic-resistant bacteria and ARGs that threaten human health [93]. The more microplastic occurs in soil the more ARGs are developed [94]. The hydrophobic surface of MPs is a facility for bacterial cell attachment and biofilm formation compared to natural constituents of soil e.g., clay minerals [95]. Moreover, MPs aging in the soil increases affinity for ARGs binding on MP surface, thus MPs become very important vectors of microbial resistance to different groups of antibiotics [96]. In aquatic ecosystems, it is also a well-known phenomenon that microbial films are built up on MPs’ surface. More likely this process also occurs in soils and colonization of microplastic by microorganisms brings a risk of plant pathogen transfer and propagation of hazardous bacteria and fungi in soil. Following this hypothesis, the use of sewage sludge in agriculture brings additional risk of human pathogen transfer and antibiotic resistance built up by microplastics up-taken by plants and leached to groundwaters. In the study of Pham et al. [95] researchers highlighted the problem, showing that microplastics present in sewage sludge serve as hubs for ARGs and pathogens. Two emerging human pathogens, *Raoultella ornithinolytica* and *Stenotrophomonas maltophilia*, were identified in soil treated with sewage sludge and PE-MPs. With the increasing disposal of plastics in the soil the health risk related to entering human pathogens with microplastics into the food chain should not be ignored. Contaminated sewage sludge or irrigation water delivers colonized microplastic onto crops. Even if the self-abundance of the human pathogen on crop leaves is susceptible to stress and degradation, the occurrence of pathogens in biofilm formed on the MP surface is the way to protect bacterial cells and increase persistence to abiotic stresses [97]. The presence of MP particles in the soil brings also the risk of plant pathogen transfer and plastics in soil are considered reservoirs of different fungal diseases harmful to plants. Fungi are part of biofilms formed on the MP surface and many different pathogenic fungal species can be identified on MP, such as *Candida*, *Fusarium*, and *Rhodotorula* [98]. These pathogens can be easily transferred to plant root systems or with irrigation water to leaves becoming the source of infection. Microfibers are common in soil due to multisource contamination transferred with water, air, and sewage sludge. According to researchers from China, fibers have the highest potential for transferring pathogens in the soil. Fibers in soils have been indicated as vectors of various groups of bacteria including *Pseudomonas*, *Aeromonas*, *Proteobacteria*, or *Actinobacteria* [99]. High accumulation of microplastic in the soil can cause changes in the microbiome, affecting nutrient cycling, mycorrhiza, and plant metabolism. Current studies do not describe the mechanism of pathogens and virus transfer to plants with MPs; however, some authors suggest an that increase in water flow in soil, erosion processes, and wind can spread microplastic-borne pathogens in agricultural ecosystems [100].

## 10. Human Health Risk Related to Microplastic Presence in Crops

The findings of this review show that microplastic in nano sizes can be easily taken up by plants and transferred to other elements of the food chain. There is evidence that plastic nanoparticles can accumulate in plant tissues, including stems, leaves, or fruits disturbing metabolic pathways which in consequence can lead to decreased yields and nutrition deficiency in crops. Moreover, microplastics in the soil are considered vectors of potentially toxic contaminants e.g., heavy metals, PAHs, or antibiotics affecting crop quality and causing concern about human and animal health. In the near future with the increasing deposition of microplastic in the environment not only the yields will be affected, but also the quality and nutritional value of crops will decrease contributing to the hidden hunger phenomena in many even well-developed countries. The detection of micro/nanoplastic absorption and translocation within plants currently [99] has a multitude of challenges and complexities. There is an urgent need to develop easily applicable methods to asses micro-/nano-plastic contamination in crops. Techniques such as Fourier transform infrared spectroscopy (FTIR), Raman spectroscopy, and Scanning Electron Microscopy (SEM) combined with Energy Dispersive X-ray Spectroscopy (EDX) are used to identify and measure microplastics in food samples [101]. However, extraction of microplastic from plant tissues is much more difficult compared to other environmental samples, as methods involving filtration or density separation are not suitable in terms of separation of plastic particles from plant tissues [13]. Micro/nanoplastics can be translocated through different parts of the plant, e.g., from roots to stems and leaves, making it challenging to track their movement and distribution within the plant system [102]. Recent advancements in machine learning and computer vision technologies like learning models and hyperspectral imaging show promise in detecting microplastics, in food and agricultural products thereby improving monitoring capabilities [103]. Despite the progress in technology, there remains a necessity for a risk assessment framework that covers hazard evaluation, exposure estimation, and health risk analysis to gain an understanding of the impacts of MP contamination [104]. Microplastic particles found in nature form a continuum of various shapes, sizes, polymer properties, and chemical characteristics, thus the framework related to risk assessment of microplastic presence in the environment should aim to maintain the complexity of microplastic [105]. In addition, exposure to various groups of chemicals present on microplastic surface also should be considered, as for various groups of organism microplastic itself might not possess any toxic effects but the presence of multiple groups of toxic compounds might cause toxicity and risk [106]. Understanding the specific mechanisms and determining the fate of micro/nanoplastics in plants are challenging experimentally because plant tissue is essentially a complex mix of biopolymers and many conditions affect micro/nanoplastic uptake and transport in plants [13]. As microplastic abundance in arable soils is always related to human activity, the input of microplastic can be estimated by modelling and the risk can be calculated based on the chemical properties of microplastic present in soil. However, this method is a simplification that can lead to overestimation due to the lack of knowledge about microplastic surface changes during aging, sorption of other contaminants in soil, bioaccumulation, and other interactions that microplastic can undergo in the soil environment. Hence, it is vital to study about assessing the health risks associated with microplastics and their ability to magnify within the food chain to maintain a grasp of this matter.

## 11. Conclusions

There is substantial scientific evidence showing that plastics can be transferred to the food chain via plants. The presence of nanoplastic particles in edible plant parts, such as roots, shoots, leaves, and seeds, poses health risks due to the potential transfer of foodborne nanoparticles to the human body. The uptake of microplastics (MPs) and nanoplastics (NPs) in plants depends on several factors, including polymer properties, MP aging processes, plastic biodegradation, particle shape and size, soil properties, plant species, and contaminant concentration in soil or water. The impact of microplastics on plant processes, such as metabolic pathways of proteins and carbohydrates, nutrient and water uptake, response to abiotic stress, and plant growth, is evident. Changes and disturbances occur at various levels, including gene expression, cellular, tissue, and whole-plant levels. Additionally, microplastics in soil can increase the bioavailability of other toxic compounds, such as heavy metals and PAHs, leading to greater bioaccumulation in edible plant parts. The effect of biodegradable versus non-biodegradable microplastics varies, with biodegradable particles enhancing microbial activity and nutrient cycling, but also contributing to the uptake of toxic compounds by plants. Concerns also extend to the role of microplastics in pathogen transfer to plants and their impact on the effectiveness of pesticides.

## 12. Future Research Recommendations

With the increasing deposition of MPs/NPs from various sources, the risk to sustainable crop production and food safety is expected to rise significantly in the coming decades. It is crucial to understand how microplastics and nanoplastics are absorbed and transported through plants to develop standard procedures for food cultivation, processing, and consumption to mitigate contamination. Future research should focus on addressing the impact of emerging contaminants on plant production and food safety. Additionally, more attention should be given to the effects of biodegradable microplastics, as they may influence soil processes, such as nutrient cycling, and plant uptake of toxic compounds. Further studies are needed to explore the potential enhanced spread of plant pathogens in the presence of microplastics and their interactions with pesticides. Finally, investigating the presence of microplastics in the soil as hotspots for antibiotic resistance genes (ARGs) is urgent, as this could negatively impact human health by contributing to antibiotic resistance.

## Figures and Tables

**Table 1:** Summary of adverse effects of micro-/nano- plastic on plant germination.

Plant Specie	Polymer Type	Effect
White clover (*Trifolium repens* L.) Chinese violet cress (*Orychophragmus violaceus)* Garden balsam (*Impatiens balsamina* L.)	Polystyrene (PS)	Germination rate reduction [36]
Onion (*Allium cepa* L.)	Polystyrene (PS)	No effect on seed germination [35]
Rice (*Oryza sativa* L.)	Polystyrene (PS) Polytetrafluoroethylene (PTFE) Polyethylene and biodegradable mulch films	Germination rate reduction [41,42]
Black Gram (*Vigna mungo* L.)	Polyethylene (PE)	Germination rate reduction [38]
Tomato (*Solanum lycopersicum* L.)	Polyethylene (PE)	No effect on seed germination [38]
Garden cress (*Lepidium sativum* L.)	Polypropylene (PP), Polyethylene (PE), Polyvinylchloride (PVC)	Germination rate reduction, increase of oxidative stress (e.g., levels of hydrogen peroxide, glutathione, and ascorbic acid [32]
Faba bean (*Vicia faba* L.)	Polystyrene (PS)	Reduced root elongation after seed germination [3]
Perennial ryegrass (*Lolium perenne* L.)	Polyhydroxybutyrate and Polylactic biodegradable polymers	35–71% reduction in root and shoot elongation after germination [37]

## Data Availability

Not applicable.
